# Comparison of the Masticatory Force (with 3D Models) of Complete Denture Base Acrylic Resins with Reline and Reinforcing Materials

**DOI:** 10.3390/ma14123308

**Published:** 2021-06-15

**Authors:** Catarina Calamote, Isabel Carolina Coelho, António Sérgio Silva, José Luís Esteves, Luís Moreira, António Correia Pinto, María Cristina Manzanares-Céspedes, Tomás Escuín

**Affiliations:** 1 Department of Oral Rehabilitation, Instituto Universitário de Ciências da Saúde, 4585-116 Gandra, Portugal; maria.andrade@iucs.cespu.pt (C.C.); isabel.coelho@iucs.cespu.pt (I.C.C.); antonio.pinto@iucs.cespu.pt (A.C.P.); 2 Dental Science Department, Instituto de Investigação e Formação Avançada em Ciências e Tecnologias da Saúde, Rua Central da Gandra 1317, 4585-116 Gandra, Portugal; 3 Department of Mechanical Engineering, Faculty of Engineering, University of Porto, 4200-465 Porto, Portugal; jesteves@fe.up.pt; 4 RECI–Research in Education and Community Intervention, Escola Superior de Saúde Jean Piaget, 4405-678 Vila Nova de Gaia, Portugal; luis.moreira@gaia.ipiaget.pt; 5 Department of Pathology and Experimental Therapy, Faculty of Odontology, University of Barcelona, 08007 Barcelona, Spain; mcmanzanares@ub.edu; 6 Department of Rehabilitation and Maxillofacial Prostheses, Faculty of Odontology, University of Barcelona, 08007 Barcelona, Spain; tescuin@ub.edu

**Keywords:** acrylic denture bases, masticatory forces, reinforcement, resin, reline materials, alveolar ridge

## Abstract

The reinforcement of acrylic denture base remains problematic. Acrylic prosthesis fractures are commonly observed in prosthodontic practice and have not been reliably resolved. This study compared the resistance to masticatory force of acrylic bases of removable complete conventional prosthesis in 3D upper models. Forty acrylic base test specimens containing two types of reinforcement meshes (20 with glass fiber meshes (FIBER-FORCE^®^- Synca, Bio Composants Médicaux^TM^, Tullins, France), 20 with metal meshes (DENTAURUM^®^-Ispringen, Germany)), 20 with a conventional PMMA acrylic base (LUCITONE 199^®^-Dentsply Sirona, York, PA, USA), and 20 using a permanent soft reline material (MOLLOPLAST-B^®^-DETAX GmbH & Co. KG, Ettlingen, Germany) were tested—a total of 80 specimens. Half of the specimens were made for a low alveolar ridge and half for a high alveolar ridge. The data were analysed using one-way analysis of variance and Student’s *t*-test for independent test specimens. In the high-alveolar-ridge group, the prosthesis reinforced with the glass fiber mesh was the most resistant to fracture, while in the low-alveolar-ridge group, the non-reinforced prosthesis showed the highest resistance masticatory force. Prostheses with the permanent soft reline material showed the lowest resistance to fracture in both high and low-alveolar-ridge groups. The results show that the selection of the right reinforcement material for each clinical case, based on the height of the alveolar ridge, may help to prevent prosthesis fractures.

## 1. Introduction

Acrylic resin fracture is still an unresolved problem in removable prosthodontics, despite the well-known causes of fracture. Modifications of acrylic resins to improve specific properties include plasticisation, co-polymerisation/cross-linking, and reinforcement. Many studies have investigated the effects of cross-linking agents on the mechanical properties of acrylic resins [1]. Several studies produced high-impact resins containing a low-molecular-weight butadiene-styrene-b co-polymer; however, the exact nature of this inclusion is considered the manufacturer’s trade secret and requires extensive chemical engineering research [1,2,3,4].

The most common method of strengthening polymeric denture bases is by adding reinforcements such as metal wires or plates to the denture base polymer. However, metal reinforcing materials form weak bonds with the resin matrix and also have poor aesthetic properties. To reinforce polymethyl methacrylate (PMMA) bases, various fiber reinforcements, such as carbon and glass fibres, plyometric polyamide, and ultra-high-molecular-weight polyethylene have been studied. Glass fibres are aesthetically stable and promote and improve the flexural strength of denture base resins, so they are most commonly used. Glass fibres also have high tensile strength when subjected to the oral environment [5,6,7,8].

The reinforcement of denture base resins remains problematic, so different materials have been tested for reinforcement, such as nanoparticles and composites [6], viscose fibres [9], mica [10], juta [8] and even vegetable fibers [11].

Fitting accuracy is an important factor for denture retention. The denture base is largely responsible for providing the prosthesis with retention, stability, and support when closely adjusted to the oral mucosa. However, bone resorption is irreversible and may lead to inadequate fitting of the prosthesis. Relining is the procedure used to resurface the tissue side of the denture with a new base material to promote accurate adjustment to the denture area [10]. Acrylic relined base prostheses play an important role when applied to edematous gengival tissue that needs to be treated with innovative therapies such as diode laser treatment [12]. Relining a denture with a resilient material guarantees better distribution of the functional load on the patient’s oral mucosa and edentulous alveolar ridge. However, current products are not suited for long-term use due to various limitations such as water absorption, premature ageing, unpleasant odours, colour changes, promotion of fungi proliferation, and reduced linkage to PMMA. The resilient materials topic is currently of great interest due to the possibility of quick adjustments to the alveolar ridge. Molloplast-B^®^ is mostly used to reline dentures [10,13]. The retention of prothesis allows good distribution of occlusal contacts and prevents temporomandibular disorders [14].

This study compared the reinforcing of metal and glass fiber mesh to a permanent soft reline material, and to a simple conventional PMMA using two different alveolar ridge heights in 3D models with replica gengival mucosa, by applying a masticatory force to the samples in a TIRA test-2705 Universal Machine^®^. This was to replicate the individual clinical aspects of the healthy palate of an upper edentulous patient with all-natural lower teeth. For this study, perfect occlusion was considered. Despite all the studies available in the literature comparing the reinforced resin bases, there is no evidence of a comparison of such materials with a permanent soft reline material, given that reline is a natural future procedure to readapt and increase the lifespan of a denture.

## 2. Materials and Methods

Lucitone 199^®^ with the natural colour 688,106 (Dentsply Sirona, York, PA, USA) was used to prepare the PMMA denture base. A pre-impregnated mesh FiBER FORCE^®^ (Synca, Bio Composants Médicaux^TM^, Tullins, France) was used for the complete prostheses for glass fiber reinforcement, a stainless metal mesh from Dentaurum^®^ (Ispringen, Germany) was used for metal reinforcement, while Molloplast-B^®^ (DETAX GmbH & Co. KG, Ettlingen, Germany) was used as a permanent soft reline material (Table 1). All materials were prepared in accordance with conventional fabrication indications.

**Figure 1:**
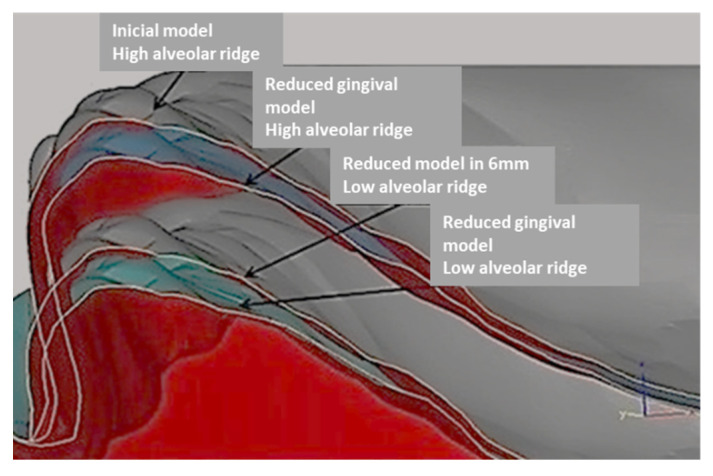
Computerised design of the four models reproduced using CAD-3D.

First, an initial upper complete edentulous model with a high alveolar ridge was selected, and then a model with a low alveolar ridge, with a 6 mm reduction, was reproduced from the initial model using the computer-assisted design software CAD-3D Buildstation^®^ (Version 5.5, 2017, Viper SLA 5000 System 3D, Darmstadt, Germany). This second model was identical to the first, except for the alveolar ridge height. These two master models were used to construct two other models for mechanical tests. Thus, there were four titanium models in total: two to create acrylic base test specimens (one with a high and one with a low alveolar ridge) and two for mechanical tests (reduced gingival models: one with a high and one with a low alveolar ridge). Figure 1 illustrates the computerised designs of the four titanium (Ti6A14V) models, all of which were reproduced using CAD-3D^®^ (Figure 1).

**Figure 2:**
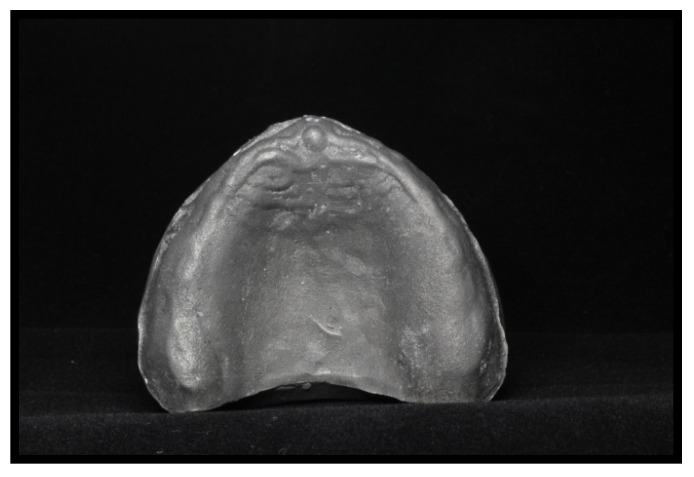
Titanium high-alveolar-ridge master model for sample construction.

**Figure 3:**
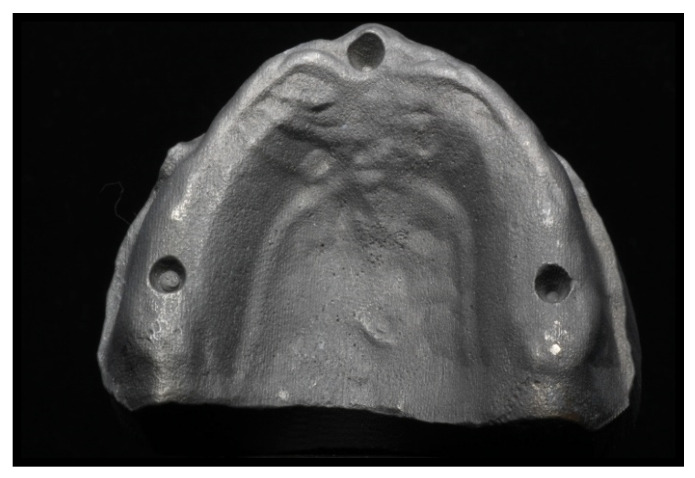
Titanium reduced gingival, high-alveolar-ridge model for mechanical tests.

**Figure 4:**
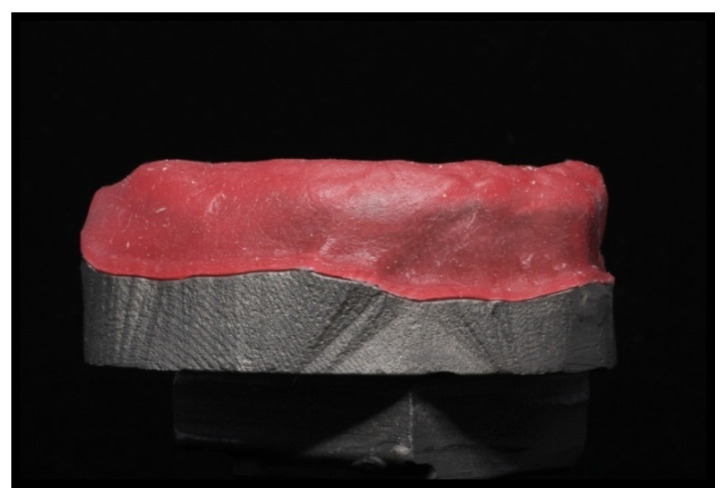
Reduced gingival, high alveolar ridge model with a healthy gingival mucosa replica made using silicone shore A-30.

Figure 2 and Figure 3 depict the initial master model and the reduced gingival model for mechanical tests, respectively, both with the high alveolar ridge. Figure 4 depicts the reduced gingival high-alveolar-ridge model for mechanical tests (with a gingival mucosa replica). The two models for mechanical tests had three small circular notches each to fit the gingival mucosa replica. Each model was reproduced using CAD-3D^®^, with the natural proportion representing the healthy gingival mucosa, built using silicone shore A-30 (Dragon Skin^®^ Smooth-On, Inc., Macungie, PA, USA). The gingival mucosa replica was reproduced with the same variability as that observed in the oral cavity: 1.5 mm at the top of the alveolar ridge, 0.5 mm at the palate, and 1 mm at the remaining locations.

**Figure 5:**
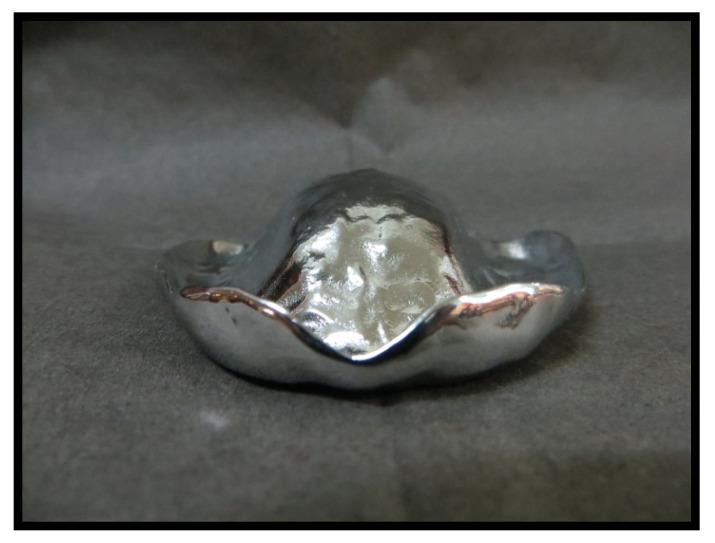
Cobalt–chromium spacer.

Eighty acrylic base test specimens were built with a cobalt–chromium spacer (cobalt–chromium dental alloy, Co-Cr) using conventional techniques according to the manufacturer’s recommendations for each reinforcement or rebase material (Figure 5).

Table 2 provides the test specimen distributions in the two study groups—the high-alveolar-ridge group and low-alveolar-ridge group (*n* = 40 each).

**Figure 6:**
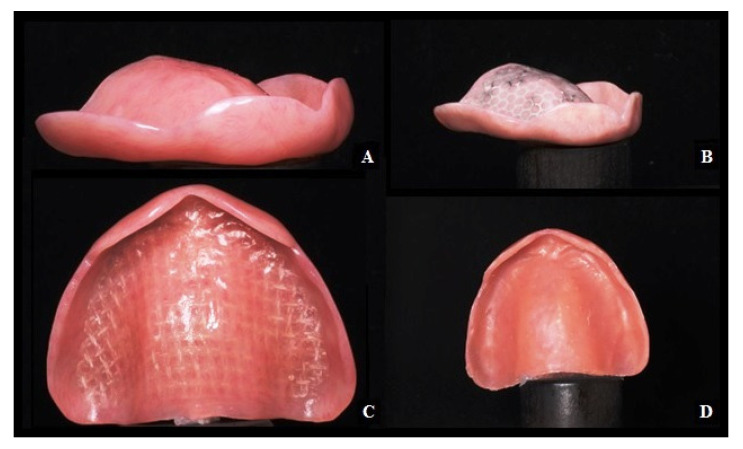
(**A**) Conventional acrylic base. (**B**) Conventional acrylic base with a metal reinforcement mesh. (**C**) Conventional acrylic base with a glass fiber mesh. (**D**) Conventional acrylic base with a permanent soft reline material.

Figure 6A–D illustrate complete upper prostheses with each type of reinforcement as well as the permanent soft reline material. The 80 acrylic base test specimens containing the two types of reinforcement meshes (20 with glass fibres, 20 with metal meshes, 20 of conventional PMMA acrylic base, and 20 using the permanent soft reline material and conventional acrylic Lucitone 199^®^) were tested. Half of the samples were built for a low alveolar ridge and half for a high alveolar ridge.

**Figure 7:**
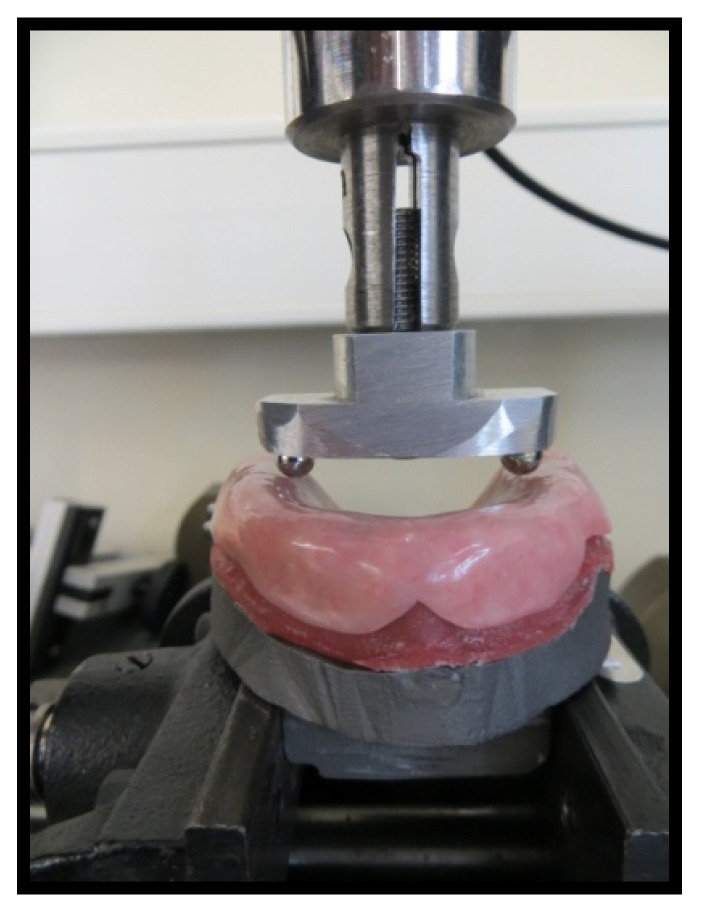
Model with a higher alveolar ridge placed on the test machine (TIRA test 2705^®^) with a special indenter comprising two spherical steel beads.

All 80 specimens were placed in a distilled water ageing bath at 37 °C for two months and then subjected to mechanical tests using a TIRA test 2705^®^ universal testing machine (DIN EN ISO/IEC 17025:2005; TIRA GmbH, Schalkau, Germany). The masticatory force of each specimen was evaluated by loading a compressive force until failure using a special indenter comprising two spherical steel beads (6 mm diameter) applied to the internal front zone of the alveolar ridge (Figure 7).

A specimen was considered to fail when the applied compressive load dropped suddenly by 25% or more of the maximum test load, corresponding to the beginning of the appearance of cracks.

The data were analysed using IBM SPSS^®^ 22.0 version, Statistics statistical software (IBM Corp., Armonk, NY, USA) using one-way analysis of variance (ANOVA) and Student’s *t*-test for independent test specimens.

## 3. Results

Table 3 provides the average values and standard deviations of the maximum masticatory forces of each type of denture base of removable, complete conventional prostheses for the low- and high-alveolar-ridge groups.

**Figure 8:**
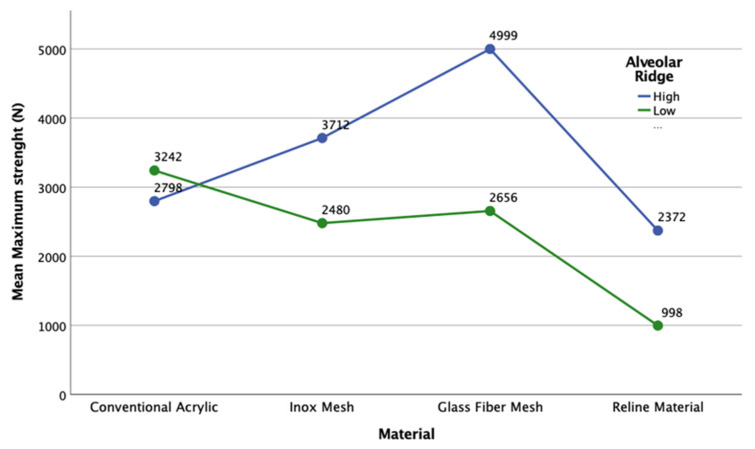
Average values of the maximum masticatory strength of each type of material in the two groups.

In the high-alveolar-ridge group, the prosthesis reinforced with the glass fiber mesh had the highest masticatory strength (4999.07 ± 0.48 N). In contrast, prosthesis reinforced with the stainless-steel mesh had approximately 26% lower (3711.57 ± 2227.96 N), the conventional acrylic prosthesis without any reinforcement had approximately 44% lower (2798.26 ± 2055.13 N), and the prosthesis with the permanent soft reline had more than 53% lower (2372.30 ± 2273.92 N) maximum masticatory force (the lowest masticatory strength in this group) (Figure 8). However, one-way ANOVA indicated that the masticatory force differences were not statistically significant (*p* = 0.394).

In the low-alveolar-ridge group, the prosthesis made of conventional acrylic without any reinforcement had the highest masticatory strength (3241.80 ± 1661.34 N). In contrast, the prosthesis reinforced with the glass fiber mesh had approximately 18% lower (2655.67 ± 1127.52 N), the prosthesis reinforced with the stainless-steel mesh had 25% lower (2480.13 ± 148.08 N), and the prosthesis with the permanent soft reline had 69% lower (997.93 ± 157.59 N) maximum masticatory strength. However, one-way ANOVA indicated that the masticatory force differences were not statistically significant (*p* = 0.118).

A comparison of the masticatory strength in the two groups showed that for each type of material, there was no statistically significant difference in the average maximum masticatory strength required for complete fracture of the upper prosthesis (*p* > 0.05). However, it is essential to consider the registered differences. Thus, in general, the results indicate that the materials used were weaker in the low-alveolar-ridge group than in the high-alveolar-ridge group. The average differences between prostheses for the high and low alveolar-ridge groups were 1231.44 N for the stainless-steel mesh, 1374.37 N for the permanent soft reline material, and 2343.40 N for the glass fiber mesh (i.e., 33, 58, and 47% reduction in masticatory strength in the low-alveolar-ridge group, respectively). In contrast, the conventional acrylic prostheses showed the reverse trend, in that the average difference between the low- and high-alveolar-ridge groups was 443.51 N (i.e., a 14% increase in masticatory strength in the low-alveolar-ridge model). The 95% confidence intervals for each type of material and the two alveolar ridge heights showed that the prosthesis reinforced with the glass fibre mesh gave the best, more homogeneous results, while the prosthesis with the reline material in the high-alveolar-ridge group gave the worst, widely variable results.

All specimens exhibited masticatory force resistance to fracture greater than 900 N and reproduced the individual clinical aspects of the healthy palate of an upper edentulous patient. All test specimens were made with a cobalt–chromium spacer to ensure that they remained similar.

## 4. Discussion

The 80 acrylic base test specimens were immersed in distilled water at 37 °C for two months to simulate artificial ageing, despite some reports that one month is sufficient to cause the diffusion of water and the softening of PMMA. The important effect of water on flexural strength occurs during the first four weeks of immersion, which reduces the flexural strength [15,16].

Cattoni et al. [17] reported that the digital method allows for great accuracy. A fully digital workflow is considered more reliable when it comes to creating an aesthetic mock-up; the digital procedure has been shown to be more accurate than the one made manually, which is much more operator-dependent and brings an increase to the chance of error, which could ultimately affect the final result. In this study, each model was designed and reproduced using digital CAD-3D^®^ to assure an excellent copy of the first model despite alveolar ridge length.

An adult with complete dentition can exert 300 to 700 N of masticatory force. The strength required for a prosthesis fracture is approximately 706 N, whereas that for a reinforced prosthesis fracture is approximately 903 N [18]. In this study, the average maximum masticatory force had a minimum limit of 998 N under the low-alveolar-ridge condition, while it had a maximum limit of 4999 N for the prosthesis reinforced with the glass fiber mesh under the high-alveolar-ridge condition. Among the Lucitone 199^®^-based samples, the lowest masticatory force (2798 N) was found under the high-alveolar-ridge condition and the highest (3242 N) under the low-alveolar-ridge condition.

Major tension or stress occurs in the alveolar crest and posterior zone such that the stress is distributed to the palate and the labial edge [19]. Ciancaglini et al. [14] analysed 25 university students with or without temporomandibular disorders and observed significantly more occlusal contacts located in the posterior than anterior area and the predominance of occlusal contacts was observed in the first molar by about 29% of patients. The good distribution of occlusal contacts avoids temporomandibular disorders. In the present study, a compression load was applied in the middle of the alveolar ridge (posterior zone left and right simultaneously) such that the height of the ridge affected the resistance of the prosthesis and to assure good distributions of occlusal contacts. Among the Lucitone 199^®^-based samples, the most resistant prosthesis was in the low-alveolar-ridge group, supporting a compression load of 3242 N; in the high-alveolar-ridge group, the prosthesis supported a compression load of 2798 N.

Many reinforced prostheses fracture due to poor adhesion between the resin base and the reinforcement material, regardless of the base and the reinforcement material used [7,20,21]. In this study, the prostheses supported a compression load of 2900 N and the reinforcement continued to support the prostheses against fracture.

Metal reinforcements have been used for many years to improve the resistance of dental prostheses, despite their aesthetic problems [22,23] and the poor adhesion of acrylic resin to metal [8]. Many studies have confirmed enhancements in the base resistance of prostheses due to the addition of metal and have studied the best method of improving the strength of metal–resin base adhesion. These methods include silanisation, sandblasting, silane addition, and linking agents; however, the ideal adhesion method has not yet been identified [24]. In the high-alveolar-ridge group, the prosthesis reinforced with the glass fiber mesh had the highest resistance to fracture when the masticatory force was applied (4999.07 ± 0.48 N); this can be explained by the good adhesion of the glass fiber mesh to the acrylic base, when compared to the other reinforcement materials.

Some studies have reported the best position and orientation for metal reinforcement [22,25,26]. In the present study, the orientation of the metal mesh was not assessed, and no adhesion was applied to the resin base. The meshes used occupied all the palate and do not have any specific orientation. They were inserted using the fabricant instructions. Notably, the masticatory forces applied on the prostheses reinforced by the stainless-steel mesh was 2480 and 3172 N under both low- and high-alveolar-ridge conditions, respectively. Similar to the findings of Balch et al. [23], the present study confirmed that the use of a metal mesh alone increases the resistance of acrylic prostheses.

Many types of fibers have been used to reinforce PMMA resin prostheses because fibres have better aesthetics [27,28]; Takahashi et al. [16]; Yu et al. [7], and many studies support the planning of the orientation, position, volume, stress, and degree of resin impregnation in the construction of a prosthesis reinforced with fiber; more importantly, these factors can influence the resistance of the resin base [2,29]. In any of these studies, the expected reinforcement is real, but no one has reached such a wide scale of masticatory forces, nor have they been used in 3D models, especially accompanied by the use of the replica of gengival mucosa.

In the present study, glass fiber was impregnated with resin to promote adhesion. In addition, because we used a glass fiber mesh, the problems associated with the position, volume, length, and orientation of the glass fibers were eliminated. The test specimens were similar in size and volume due to the use of a cobalt–chromium spacer during construction. Therefore, the reinforced prosthesis included less resin in the acrylic base because of the areas occupied by the reinforcing material. Similar to prostheses reinforced with a metal mesh, in addition to enduring a masticatory strength of 2656 N under the low-alveolar-ridge condition and 4999 N under the high-alveolar-ridge condition, glass fibers also held the fractured prosthesis together.

According to many authors, the simple use of glass fibers increases the masticatory force of PMMA resin bases [5,9,30,31,32,33,34,35]. In this study, the prosthesis reinforced with a glass fiber mesh showed the highest masticatory force under the high-alveolar-ridge condition (4999 N).

Denture reline materials have been used in dentistry for more than a century, and these materials continue to play an important role in modern prosthodontics. Soft reline materials are for temporary use, and hard ones are for posterior adaptations of the alveolar ridges. Many reline materials have been used with varying levels of success; however, they have some limitations, including volumetric changes, abrasion, colour instability, water absorption, and solubility, in addition to problems of denture weakening. The relining of a complete prosthesis achieves the best distribution of the functional load on the patient’s oral mucosa and edentulous alveolar ridge. Reline materials allow the restoration of oral conditions that improve prosthesis retention. Reline materials function as shock absorbers between the alveolar ridge and the prosthesis [36]. Despite these advantages of reline materials, they are not widely used, because they have limited usage duration as they lose resilience and linkage to the prosthesis, absorb odours, permit fungi proliferation, and increase prosthesis weakness [13]. In this study, test specimens with permanent soft reline materials showed the lowest masticatory resistance under both high- and low-alveolar-ridge conditions.

Some studies have reported that the reline material Molloplast-B^®^ has good linkage with acrylic prostheses, and the results of the present study support this conclusion [36,37].

## 5. Conclusions

The prosthesis resistance to masticatory forces varies depending on the alveolar ridge height. In addition, the masticatory resistance is not always greater when the denture is reinforced with a mesh. Furthermore, the addition of a permanent soft reline material results in the lowest masticatory resistance under both low- and high-alveolar-ridge conditions.

Stainless steel and glass fiber mesh induced similar trends of masticatory strength in the low-alveolar-ridge prosthesis. Glass fiber mesh has a high value as a reinforcement material due to its excellent masticatory strength performance. Among the reinforcement materials tested, with the exception of conventional acrylic, lower masticatory strength was observed more in the low-alveolar-ridge than in the high-alveolar. The addition of a permanent soft reline material to PMMA decreased the resistance to masticatory strength. Furthermore, the tested meshes held the prostheses together after fracture.

The results show that selection of the right reinforcement material for each clinical case based on the height of the alveolar ridge may help to prevent prosthesis fracture.

## Figures and Tables

**Table 1:** Trademarks of the selected materials.

Material	Trademark	Lot	Characteristics
PMMA	Lucitone 199^®^ by Dentsply Sirona	110,517; York, PA, USA	Terminal acrylic Conventional
Glass fibre mesh	FiBER FORCE^®^ by Synca	13,176; Bio Composants Médicaux^TM^, Tullins, France	Pre-impregnated
Metal inox mesh	Dentaurum^®^	440,990; Ispringen, Germany	-
Reline material	Molloplast B^®^	160,526; Ettlingen, Germany	Permanent soft reline

**Table 2:** Test specimen distribution and groups of materials.

Material	High Alveolar Ridge	Low Alveolar Ridge
Lucitone 199^®^	10	10
FiBER FORCE^®^	10	10
Metal mesh Dentaurum^®^	10	10
Permanent soft reline Molloplast-B^®^	10	10
	*n* = 40	*n* = 40

**Table 3:** Average maximum masticatory strength of each type of material for low- and high-alveolar-ridge groups.

Material	High Alveolar Ridge		Low Alveolar Ridge			*p*-Value ^†^
	Mean (N)	SD	Mean (N)	SD	Difference * (N)	
Lucitone 199^®^	2798.28	2055.13	3241.80	1661.34	−443.51	0.786
Metal mesh Dentaurum^®^	3711.57	2227.96	2480.13	148.08	1231.44	0.440
Glass fibre mesh FiBER FORCE^®^	4999.07	0.48	2655.67	1127.52	2343.40	0.069
Permanent soft reline Molloplast-B^®^	2372.30	2273.92	997.93	157.59	1374.37	0.405
*p*-Value ^‡^	0.394	-	0.118	-	-	-

* Difference between means: high alveolar ridge vs. low alveolar ridge. ^†^ Student’s *t*-test for two independent samples. ^‡^ One-way analysis of variance. SD: standard deviation.

## Data Availability

No data other than that shown in the manuscript has been reported.

## References

[1] Vallittu P.K. (1999). Flexural properties of acrylic resin polymers reinforced with unidirectional and woven glass fibers. J. Prosthet. Dent..

[2] Praveen B., Babaji H.V., Prasanna B.G., Rajalbandi S.K., Sheeharsha T.V., Prashant G.M. (2014). Comparison of Impact strength and fracture morphology of different heat Cure denture acrylic resins: An in vitro study. J. Int. Oral Health.

[3] De Sá J., Vieira F., Aroso C.M., Cardoso M., Mendes J.M., Silva A.S. (2020). The influence of saliva pH on the fracture resistance of three complete denture base acrylic resins. Int. J. Dent..

[4] Silva A.S., Carvalho A., Barreiros P., de Sá J., Aroso C., Mendes J.M. (2021). Comparison of fracture resistance in thermal and self-curing acrylic resins—An in vitro study. Polymers.

[5] Jagger D., Harrison A., Vowles R., Jagger R. (2001). The effect of the addition of surface treated chopped and continuous poly (methyl methacrylate) fibres on some properties of acrylic resins. J. Oral Rehabil..

[6] Tsue F., Takahashi Y., Shimizu H. (2007). Reinforcing effect of glass-fiber-reinforced composite on flexural strength at the proportional limit of denture base resin. Acta Odontol. Scand..

[7] Yu S.-H., Cho H.-W., Oh S., Bae J.-M. (2015). Effects of glass fiber mesh with different fiber content and structures on the compressive properties of complete dentures. J. Prosthet Dent..

[8] Alla R.K., Sajjan S., Alluri V.R., Ginjupalli K., Upadhya N. (2013). Influence of fiber reinforcement on the properties of denture base resins. J. Biomater. Nanobiotechnol..

[9] Dogan O.M., Bolayir G., Keshin S., Dogan A., Bek B. (2008). The evaluation of some flexural properties of a denture base resin reinforced with various aesthetic fibers. J. Mater. Sci. Mater. Med..

[10] Mansour M.M., Wagner W.C., Chu T.G. (2013). Effect of MICA reinforcement on the flexural strength and microhardness of polymethyl methacrylate denture resin. J. Prosthodont..

[11] Xu J., Li Y., Yu T., Cong L. (2013). Reinforcement of denture base resin with short vegetable fiber. Dent. Mater..

[12] Polizzi E., Tetè G., Targa C., Gastaldi G. (2020). Evaluation of the effectiveness of the use of the Diodo Laser in the reduction of the volume of the edematous gingival tissue after causal therapy. Int. J. Environ. Res. Public Health.

[13] Mohammed S.A., Hassen K.S. (2011). Evaluation of some properties of four silicon based soft dentures reliner materials. J. Bagh. Coll. Dent..

[14] Ciancaglini R., Gherlone E.F., Redaelli S. (2002). The distribution of occlusal contacts in the intercuspal position and temporomandibular disorder. J. Oral Rehabil..

[15] Vojvodic D., Komar D., Schauperl Z., Celebic A., Mehulic K., Zabarovic D. (2009). Influence of different glass fiber reinforcements on denture base polymer strength (Fiber reinforcements of dental polymer). Med. Glass.

[16] Takahashi Y., Yoshida K., Shimizu H. (2011). Effect of location of glass fiber-reinforced composite reinforcement on the flexural properties of a maxillary complete denture in vitro. Acta Odontol. Scand..

[17] Cattoni F., Tetè G., Calloni A.M., Manazza F., Gastaldi G., Capparè P. (2019). Milled versus moulded mock-ups based on the superimposition of 3D meshehes from digital oral impressions: A comparative in vitro study in the aesthetic area. BMC Oral Health.

[18] Prombonas A.E., Vlissidis D.S., Maria P.A., Nikolas P.A. (2012). The stress state of the fraenal notch region in complete upper dentures. Med. Eng. Phys..

[19] Ates M., Çilingir A., Sülün T., Sunbuloglu E., Bozdag E. (2006). The effect of occlusal contact localization on the stress distribution in complete maxillary denture. J. Oral Rehabil..

[20] Hirajima Y., Takahashi H., Minakushi S. (2009). Influence of a denture strengthener on the deformation of a maxillary complete denture. Dent. Mater. J..

[21] Vogdani M., Bagheri R., Khaledi A.A.R. (2012). Effects of aluminum oxide addition on the flexural strength, surface hardness, and roughness of heat-polymerized acrylic resin. J. Dent. Sci..

[22] Foo S.H., Lindquist T.J., Aquilino S.A., Schneider R.L., Williamson D.L., Boyer D.B. (2001). Effect of polyaramid fiber reinforcement on the strength of 3 Denture Base polymethyl methacrylate resin. J. Prosthodont..

[23] Balch J.H., Smith P.D., Marin M.A., Cagna D.R. (2013). Reinforcement of a mandibular complete denture with internal metal framework. J. Prosthet. Dent..

[24] Teraoka F., Nakagawa M., Takahashi J. (2001). Adaptation of Acrylic dentures reinforced with metal wire. J. Oral Rehabil..

[25] Shimizu H., Mori N., Takahashi Y. (2008). Use of metal conditioner on reinforcement wires. N. Y. State Dent. J..

[26] Yoshida K., Takashashi Y., Shimizu H. (2011). Effect of embedded metal reinforcements and their location on the fracture resistance of acrylic resin complete dentures. J. Prosthodont..

[27] Jagger D.C., Harrison A., Jandt K.D. (1999). The reinforcement of dentures-review. J. Oral Rehabil..

[28] Chen S.Y., Liang W.M., Yen P.S. (2001). Reinforcement of acrylic denture base resin by incorporation of various fibers. J. Biomed. Mater. Res..

[29] Bertassoni L.E., Marshall G.W., Souza E.M., Rached R.N. (2008). Effect of pre and post polymerization on flexural strength and elastic modules of impregnated, fiber-reinforced denture base acrylic resins. J. Prosthet. Dent..

[30] Kanie T., Arikawa H., Fujii K., Ban S. (2003). Impact strength of acrylic denture base resin reinforced with woven glass fiber. Dent. Mater. J..

[31] Koroglu A., Ozdemir T., Pamir A.D., Usanmaz A. (2012). Residual acrylic monomer content of denture base resins with different fiber systems. J. Appl. Polym. Sci..

[32] Takahashi Y., Yoshida K., Shimizu H. (2012). Fracture resistance of maxillary complete dentures subjected to long term water immersion. Gerodontology.

[33] Yu S.-H., Lee Y., Oh S., Cho H.-W., Oda Y., Bae J.-M. (2012). Reinforcing effects of different fibers on denture base resin based on the fiber type, concentration, and combination. Dent. Mater. J..

[34] Cheng Y.Y., Cheung W.L., Chow T.W. (2010). Strain analysis of maxillary complete denture with three-dimensional finite element method. J. Prosthet. Dent..

[35] Cheng Y.Y., Li J.Y., Cheung W.L., Chow T.W. (2010). 3D FEA of high-performance polyethylene fiber reinforced maxillary dentures. Dent. Mater..

[36] Denir H., Dogan A., Dogan O.M., Keskin S., Bolayir G., Soygun K. (2011). Peel bond strength of two silicone soft liners to a heat-cured denture base resin. J. Adhes. Dent..

[37] Hatamleh M.M., Maryan C.J., Silikas N., Watts D.C. (2010). Effect of net fiber reinforcement surface treatment on soft denture liner retention and longevity. J. Prosthodont..

